# Endocardite de Prótese Valvular Aórtica por *Neisseria Elongata* após Procedimento de Bentall: Quando a Imagem Multimodal é Chave para o Diagnóstico

**DOI:** 10.36660/abc.20200706

**Published:** 2021-05-06

**Authors:** Mariana Brandão, Pedro Gonçalves-Teixeira, Pedro Ribeiro Queirós, Nuno Dias Ferreira, Marco Oliveira

**Affiliations:** 1 Centro Hospitalar de Vila Nova de Gaia Espinho EPE Vila Nova de Gaia Portugal Centro Hospitalar de Vila Nova de Gaia Espinho EPE, Vila Nova de Gaia - Portugal

**Keywords:** Insuficiência da Valva Aórtica/cirurgia, Aneurisma Aórtico, Próteses Valvulares Cardíacas, Endocardite, Neisseria Elongata, Tomografia Computadorizada/métodos, Ecocardiografia/métodos

Homem diabético de 65 anos com procedimento de Bentall prévio e prótese valvar aórtica mecânica apresentou febre e dor abdominal, juntamente com sopro sistólico (III/VI) e marcadores inflamatórios elevados. A tomografia computadorizada (TC) abdominal revelou infarto esplênico. Ecocardiograma transesofágico (ETE) foi negativo para vegetações. Diante da suspeita persistente de endocardite infecciosa (EI) com embolia periférica, iniciou-se terapia antimicrobiana empírica. Posteriormente, o paciente apresentou bloqueio atrioventricular total, necessitando de estimulação transvenosa temporária. Posteriormente, implantou-se um marca-passo epicárdico.

**Figura 1:**
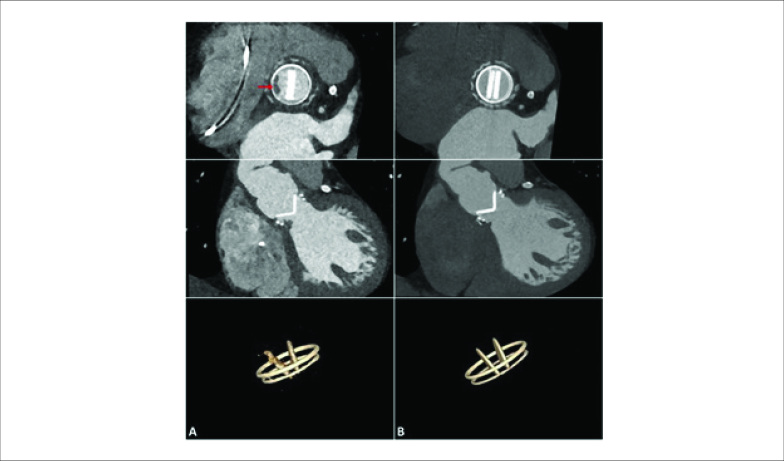
1A) TC cardíaca na admissão mostrando vegetação na valva aórtica e alterações inflamatórias na fibrosa intervalvar e espaços septais interatriais. 1B) TC cardíaca na alta com tecido inflamatório residual.

**Figura 2:**
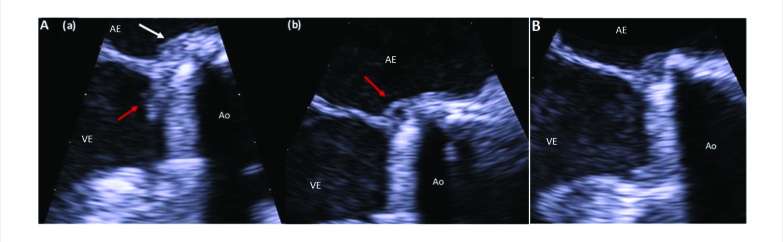
2A) Ecocardiograma transesofágico, corte esôfago médio eixo longo da valva aórtica. (a) Vegetação (seta vermelha) e infiltrado inflamatório (seta branca). (b) Abscesso anular (seta vermelha). Ao: aorta ascendente; AE: átrio esquerdo; VE: ventrículo esquerdo. 2B) Ecocardiograma transesofágico na alta, corte esôfago médio eixo longo da valva aórtica. Não há visualização de vegetação ou abscesso. Ao: aorta ascendente; AE: átrio esquerdo; VE: ventrículo esquerdo.

Na ocasião, a TC cardíaca revelou uma massa hipoatenuante de formato irregular apensa ao lado ventricular do anel de sutura protético, compatível com vegetação (Figura 1A), interferindo na abertura normal de um dos discos da prótese (Vídeo 1). Um novo ETE também mostrou uma pequena vegetação altamente móvel e um abscesso anular na prótese aórtica (Figura 2). As hemoculturas foram positivas para *Neisseria elongata*, confirmando o diagnóstico de endocardite de válvula protética (EVP); terapia antimicrobiana foi adaptada. Apesar da melhora precoce, o paciente posteriormente apresentou ataxia “de novo” e a TC de crânio revelou infarto no território vertebrobasilar direito. Novas culturas continuaram negativas e os níveis de coagulação estavam dentro da faixa terapêutica. Uma pequena vegetação persistia na TC cardíaca e no ETE, e infiltrado inflamatório tornou-se aparente na cortina mitro-aórtica.

**Vídeo 1:**
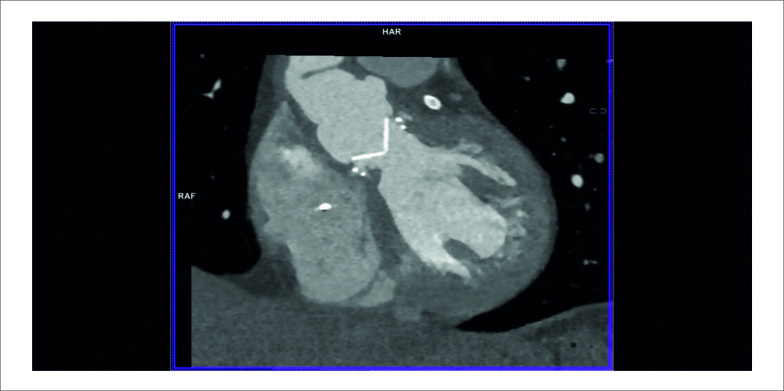
URL: http://abccardiol.org/supplementary-material/2021/11605/2020-0706-video1.mp4

O paciente foi recusado para cirurgia devido ao risco proibitivo de reoperação, e uma estratégia conservadora foi adotada após discussão em *Heart Team*. Após oito semanas de antibioterapia, alcançou-se remissão clínica e laboratorial. A TC revelou uma prótese normofuncionante (Vídeo 2) e os achados patológicos previamente observados estavam ausentes (VFigura 1B). As vegetações não eram mais evidentes ao ETE (Figura 3).

**Vídeo 2:**
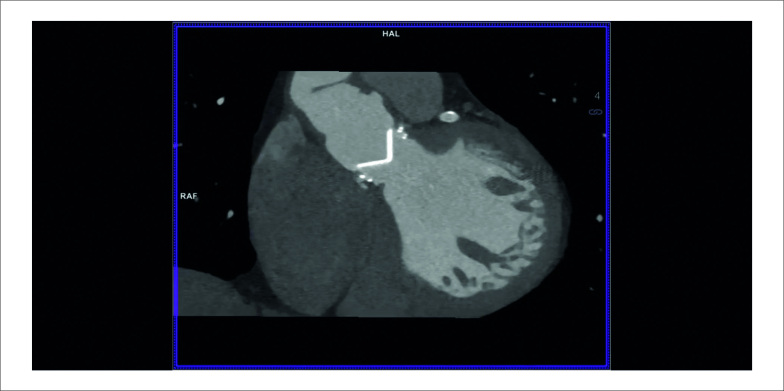
URL: http://abccardiol.org/supplementary-material/2021/11605/2020-0706-video2.mp4

Após um ano de seguimento, o doente permaneceu assintomático, sem sinais ecocardiográficos ou laboratoriais de recidiva.

Tanto quanto sabemos, este é o primeiro relato de caso de EVP em paciente com procedimento de Bentall prévio devido a *Neisseria elongata*. Destacamos a importância da imagem multimodal, principalmente quando o diagnóstico permanece incerto após avaliação ecocardiográfica inconclusiva. Em última análise, o diagnóstico se baseou nos achados da TC, incluído como um dos critérios major diagnósticos nas diretrizes mais recentes de endocardite.^1^ A TC tem excelente resolução espacial e permite a visualização detalhada da anatomia paravalvar e suas complicações, com menos artefato de sombra da prótese.^2^

Apesar das indicações cirúrgicas óbvias, o paciente foi tratado com sucesso com uma estratégia conservadora (controversa). Embora a remoção e a substituição do material protésico fossem tradicionalmente consideradas obrigatórias, se a intervenção não for viável, os pacientes devem ser tratados com antibioterapia prolongada.^3^ Diversas séries, incluindo o registro ESC-EORP EURO-ENDO,^4^ declararam a discrepância entre as indicações cirúrgicas orientadas por diretrizes e a prática real, em grande parte explicada por pacientes cada vez mais complexos, com mais comorbidades e intervenções prévias com material protésico intracardíaco. Este caso é ilustrativo dos desafios atuais envolvidos no diagnóstico e tratamento de EVP, onde o tratamento conservador pode, ocasionalmente, ser bem-sucedido e ser a única opção aceitável.
