# Supplementary material for: Endocardite de Prótese Valvular Aórtica por Neisseria Elongata após Procedimento de Bentall: Quando a Imagem Multimodal é Chave para o Diagnóstico
Source: Arq Bras Cardiol. 2021 May 6;116(5):1023–6. [Article in Portuguese] doi: 10.36660/abc.20200706 (PMC8121472; doi:10.36660/abc.20200706)
Supplement: Translation

# Translation: Prosthetic Aortic Valve Endocarditis by *Neisseria Elongata* after Bentall Procedure: When Multimodality Imaging is Key to Diagnosis

**Authors:** Mariana Brandão, Pedro Gonçalves-Teixeira, Pedro Ribeiro Queirós, Nuno Dias Ferreira, Marco Oliveira

**Affiliations:** 1 Vila Nova de Gaia Portugal Centro Hospitalar de Vila Nova de Gaia Espinho EPE, Vila Nova de Gaia – Portugal

**Keywords:** Infective endocarditis, Neisseria elongata, Bentall procedure, Prosthetic valve, Multimodality imaging

A diabetic 65-year-old male with previous Bentall procedure and mechanical aortic valve prosthesis presented with fever and abdominal pain, along with systolic murmur (III/VI) and elevated inflammatory markers. Abdominal computed tomography (CT) revealed splenic infarction. Transesophageal echocardiogram (TEE) was negative for vegetations. Given the persistent suspicion of infective endocarditis (IE) with peripheral embolism, empirical antimicrobial therapy was initiated. The patient subsequently presented with complete atrioventricular block, requiring temporary transvenous pacing. An epicardial pacemaker was later implanted.

At the time, cardiac CT revealed an irregular-shaped hypoattenuating mass attached to the ventricular side of the prosthetic suture ring, consistent with vegetation (Figure 1A), interfering with the normal opening of one of the prosthesis discs (Video 1). Repeat TEE also showed a small highly mobile vegetation and an annular abscess in the prosthetic aortic valve (Figure 2). Blood cultures were positive for *Neisseria elongata*, confirming the diagnosis of prosthetic valve endocarditis (PVE); antimicrobial therapy was tailored. Despite early improvement, the patient later presented with de novo ataxia and brain CT revealed infarction in the right vertebrobasilar territory. New sets of cultures remained negative and coagulation levels were within therapeutic range. A small vegetation persisted on cardiac CT and TEE, and inflammatory infiltrate was apparent at the mitro-aortic curtain.

**Figure 1:** 1A) Cardiac CT scan at admission showing vegetation in the aortic valve and inflammatory changes in the intervalvular fibrosa and inter-atrial septal spaces. 1B) Cardiac CT scan at discharge with residual inflammatory tissue.

**Figure 2:** 2A) Transesophageal echocardiogram, midesophageal long-axis aortic valve plane. (a) Vegetation (red arrow) and inflammatory infiltrate (white arrow). (b) Annular abscess (red arrow). Ao: ascending aorta; LA: left atrium; LV: left ventricle. 2B) Transesophageal echocardiogram at discharge, midesophageal long-axis aortic valve plane. No vegetation or abscess visible. Ao: ascending aorta; LA: left atrium; LV: left ventricle.

**Video 1:** URL: http://abccardiol.org/supplementary-material/2021/11605/2020-0706-video1.mp4

The patient was refused for surgery due to the prohibitively high risk of re-operation, and a conservative strategy was pursued after Heart Team discussion. Following eight weeks of antimicrobial therapy, clinical and laboratory remission were achieved. CT scan disclosed a normally functioning prosthesis (Video 2) and the previously observed pathological findings were absent (Figure 1B). Vegetations were no longer evident on TEE (Figure 3).

**Video 2:** URL: http://abccardiol.org/supplementary-material/2021/11605/2020-0706-video2.mp4

The patient remained asymptomatic at 1-year follow-up, without echocardiographic or laboratory signs of recurrence.

This is, to our knowledge, the first case report of PVE in a patient with previous Bentall procedure due to *Neisseria elongata*. We highlight the importance of multimodality imaging, particularly when diagnosis remains uncertain after inconclusive echocardiographic evaluation. The diagnosis ultimately relied on CT findings, included as a major diagnostic criterion in the latest endocarditis guidelines.^1^ CT has excellent spatial resolution and enables detailed visualization of paravalvular anatomy and complications, with less artifact and shadowing from the prosthesis.^2^

Despite the obvious surgical indications, the patient was successfully treated with a conservative (controversial) strategy. Although removal and replacement of prosthetic material was traditionally considered mandatory, if intervention is not feasible, patients should be treated with prolonged antibiotic therapy.^3^ Multiple series, including the ESC-EORP EURO-ENDO registry,^4^ have stated the discrepancy between guideline-directed surgical indications and actual practice, largely explained by evermore complex patients, with more co-morbidities and previous interventions with intracardiac prosthetic material. This case is illustrative of the current challenges involved in the diagnosis and management of PVE, where conservative treatment may sometimes prove successful and the only acceptable option.
